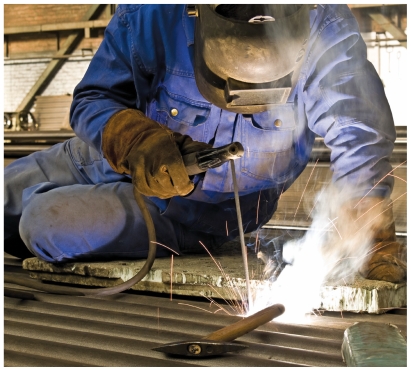# A Compendium of Challenges: Assessing the State of the Science on Occupational Carcinogens

**DOI:** 10.1289/ehp.118-a444b

**Published:** 2010-10

**Authors:** M. Nathaniel Mead

**Affiliations:** **M. Nathaniel Mead**, a science writer living in Durham, NC, has written for *EHP* since 2002

Uncertainties abound about the adverse health effects of exposure to carcinogens found in today’s workplaces. Even with substantial toxicologic evidence of carcinogenicity, cancer risks for humans often remain inconclusive, thus delaying regulatory action and the search for safer alternatives. A new systematic review by the International Agency for Research on Cancer (IARC) identifies research gaps and needs for 20 agents prioritized for review on the basis of evidence of widespread human exposures and potential carcinogenicity in animals or humans [*EHP* 118(10):1355–1362; Ward et al.].

Drawing from an international collaboration by 25 health and research agencies and institutions, the report summarizes recommendations and broaches key topics pertaining to several chemicals, metals, dusts, and physical agents for which there is widespread human exposure, predominantly in occupational settings. The authors emphasize that carcinogenic agents can act through multiple pathways and mechanisms, including oxidative stress, epigenetic mechanisms, and immuno- and hormonal modulation. They then discuss overarching issues pertinent to the study of these mechanisms. For example, regarding the validation of oxidative stress biomarker assays, they write, “Research is needed to examine the relationship between exposure to toxic agents and oxidative stress biomarkers, and between these biomarkers and risk of cancer, while controlling for the many individual factors that contribute to oxidative stress.”

Concerning genetic susceptibility to carcinogenic exposures, the authors caution that stable and reproducible associations are few. Examining genetic polymorphisms related to carcinogen metabolism and/or DNA repair may aid the identification of higher cancer risks in susceptible subgroups and clarify the role of specific agents in mixed exposures. Nonetheless, the magnitude of such associations may be modest and could entail multiple genes or metabolic pathways—thus making them hard to detect.

The report deals with only a fraction of the potentially carcinogenic agents found in today’s workplaces, most of which have sufficient evidence of carcinogenicity in animals but limited evidence for carcinogenicity in humans. Because of a paucity of well-designed animal bioassays and human studies, insufficient evidence exists to evaluate animal or human carcinogenicity for most other agents. The report ends on a somber note, noting that substantial challenges for the study of environmental carcinogens remain, including a recent decline in funding for occupational cancer research, and that fewer scientists are entering the fields of epidemiology, toxicology, and exposure assessment.

## Figures and Tables

**Figure:**